# Correction: ADAD2 regulates heterochromatin in meiotic and post-meiotic male germ cells via translation of MDC1

**DOI:** 10.1242/jcs.261751

**Published:** 2023-12-18

**Authors:** Lauren G. Chukrallah, Aditi Badrinath, Gabrielle G. Vittor, Elizabeth M. Snyder

This Correction updates and replaces the Expression of Concern (doi:10.1242/jcs.260435) relating to *J. Cell Sci.* (2022) **135**, jcs259196 (doi:10.1242/jcs.259196).

Journal of Cell Science was made aware of issues with western blot presentation by a reader of the above article. After examination of the article and the original data, and with the cooperation and assistance of the corresponding author Dr Elizabeth M. Snyder, issues with figure preparation and data management affecting multiple figures were identified. The journal referred these matters to Dr Snyder's institute, Rutgers University, for investigation. An Inquiry Panel convened by Rutgers University recommended a research misconduct investigation was not necessary. Given that the Rutgers University Inquiry Panel made no finding of research misconduct, the journal is publishing this Correction to address the blot presentation issues identified in the article. This Correction also adds details of reuse of control data within the article to the relevant figure legends and the Materials and Methods.

In Fig. 1D, the HP1α blot for samples from isolated cells was cropped incorrectly, and the GAPDH blot for the 21 dpp samples was vertically inverted. Additionally, the images shown as HP1β blots for 21 dpp and adult samples were images of the same western blot; they have been replaced with images from technical replicate experiments in the corrected figure, and the quantification of HP1β protein band intensity has been updated using the corrected images. The corrected and original panels are shown below. The figure legend has been updated to add details of data reuse.

**Fig. 1D (corrected panel).**
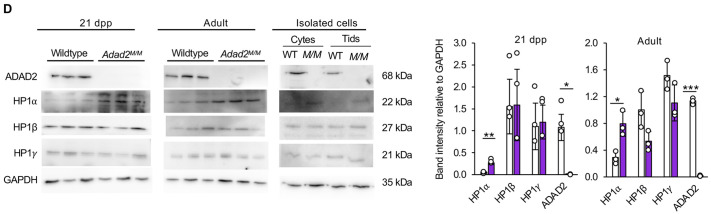
***Adad2* mutant meiotic spermatocytes and post-meiotic spermatids have abnormal localization and increased levels of heterochromatin.** (D) Western blot of HP1 proteins in 21 dpp and adult whole-testis lysate from wild-type and *Adad2^M/M^* samples (three biological samples per genotype) and from enriched pools of adult wild-type and *Adad2^M/M^* spermatocytes and spermatids (pooled across three biological samples per genotype). ADAD2 and GAPDH are genotype and loading controls, respectively. The 21 dpp ADAD2 genotype control and GAPDH loading control blots are also shown for the same protein panel in Fig. 3A, Fig. S5B,D and Fig. S6D. The adult ADAD2 genotype control and GAPDH loading control blots are also shown for the same protein sample panel in Fig. S5D. Quantification of band intensity confirms a significant increase of HP1α in mutants at both ages.

**Fig. 1D (original panel).**
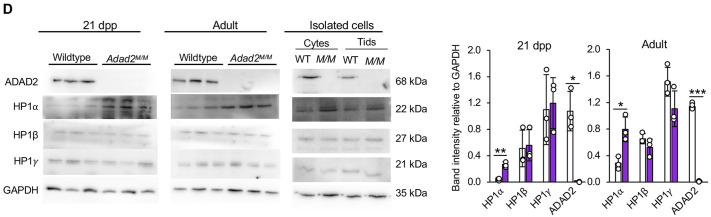
***Adad2* mutant meiotic spermatocytes and post-meiotic spermatids have abnormal localization and increased levels of heterochromatin.** (D) Western blot of HP1 proteins in 21 dpp and adult whole-testis lysate from wild-type and *Adad2^M/M^* samples (three biological samples per genotype) and from enriched pools of adult wild-type and *Adad2^M/M^* spermatocytes and spermatids (pooled across three biological samples per genotype). ADAD2 and GAPDH are genotype and loading controls, respectively. Quantification of band intensity confirms a significant increase of HP1α in mutants at both ages.

In Fig. 1E, the H3K9me3 blot for samples from isolated cells was incorrectly cropped and horizontally inverted. The corrected and original panels are shown below. The figure legend has been updated to add details of data reuse.

**Fig. 1E (corrected panel).**
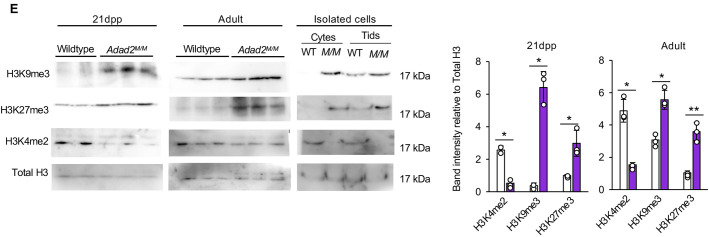
***Adad2* mutant meiotic spermatocytes and post-meiotic spermatids have abnormal localization and increased levels of heterochromatin.** (E) Western blot of select epigenetic marks in 21 dpp and adult whole-testis histone lysate from wild-type and *Adad2^M/M^* samples (*n*=2 samples for 21 dpp wild type; *n*=3 for all others) and enriched pools of adult wild-type and *Adad2^M/M^* spermatocytes and spermatids (pooled across three biological samples per genotype) with total histone 3 (H3) as a loading control. The 21 dpp total H3 loading control blot is also shown for the same protein sample panel in Fig. S6A. Quantification of band intensity shows increased heterochromatin, measured by H3K9me3 and H3K27me3, and decreased euchromatin, measured by H3K4me2.

**Fig. 1E (original panel).**
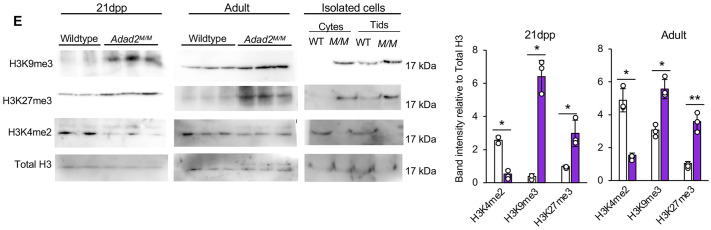
***Adad2* mutant meiotic spermatocytes and post-meiotic spermatids have abnormal localization and increased levels of heterochromatin.** (E) Western blot of select epigenetic marks in 21 dpp and adult whole-testis histone lysate from wild-type and *Adad2^M/M^* samples (*n*=2 samples for 21 dpp wild type; *n*=3 for all others) and enriched pools of adult wild-type and *Adad2^M/M^* spermatocytes and spermatids (pooled across three biological samples per genotype) with total histone 3 (H3) as a loading control. Quantification of band intensity shows increased heterochromatin, measured by H3K9me3 and H3K27me3, and decreased euchromatin, measured by H3K4me2.

In Fig. 3A, the ADAD2 and GAPDH blots were vertically inverted, and the BRCA blot was cropped incorrectly and vertically inverted. Additionally, the legend of this panel incorrectly stated that the blots used adult testis lysate instead of 21 dpp testis lysate (as correctly reported elsewhere in the article). The figure legend has been updated to correct this error and add details of data reuse. The corrected and original panels are shown below.

**Fig. 3A (corrected panel).**
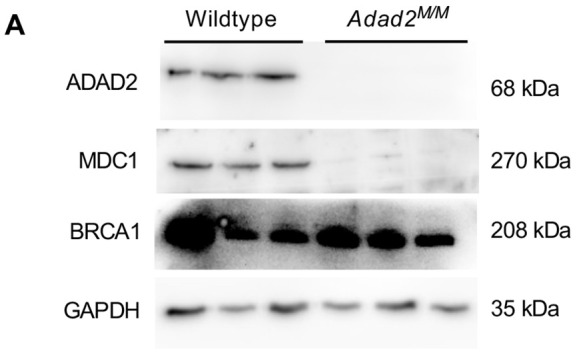
**Loss of ADAD2 results in altered abundance of two key DNA damage response proteins.** (A) Western blot of MDC1 and BRCA1 in wild-type and *Adad2^M/M^* 21 dpp testis lysate shows reciprocal impacts on protein abundance in *Adad2* mutants. GAPDH and ADAD2 blots shown as loading and genetic controls (three samples/genotype). The ADAD2 genotype control and GAPDH loading control blots are also shown for the same protein sample panel in Fig. 1D, Fig. S5B,D and Fig. S6D.

**Fig. 3A (original panel).**
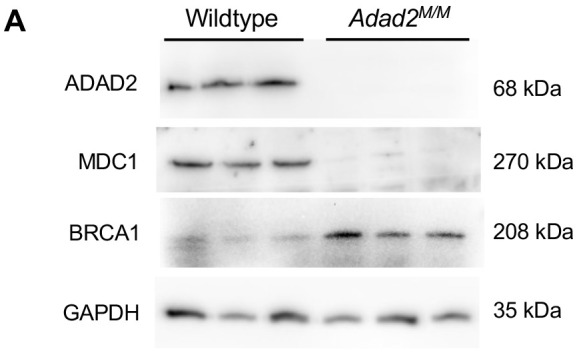
**Loss of ADAD2 results in altered abundance of two key DNA damage response proteins.** (A) Western blot of MDC1 and BRCA1 in wild-type and *Adad2^M/M^* adult testis lysate shows reciprocal impacts on protein abundance in *Adad2* mutants. GAPDH and ADAD2 blots shown as loading and genetic controls (three samples/genotype).

In Fig. S5B, the GAPDH blot shown was incorrect, and this incorrect blot was used for normalisation of the data plotted in Fig. S5C. The corrected and original panels are shown below. The figure legend has been updated to add details of data reuse.

**Fig. S5B,C (corrected panels).**
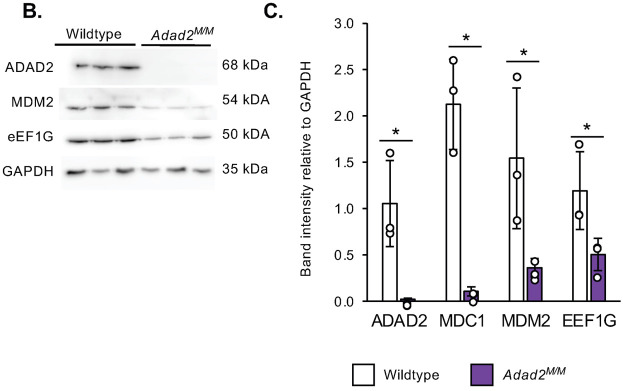
**Altered ribosome association similar to Mdc1 is observed in multiple transcripts but abundance and ribosome association is unaltered in heterochromatin remodeling genes in Adad2 mutants.** (B) Western blot against MDM2 and eEF1G in 21 dpp wildtype and mutant testes and (C) quantification of band intensity in B shows similar reduction of protein in spite of increased ribosome association. ADAD2 shown as genetic control (n=3/genotype). The ADAD2 genotype control and GAPDH loading control blots are also shown for the same protein sample panel in Fig. 1D, Fig. 3A, Fig. S5D and Fig. S6D. Plotted points represent individual samples.

**Fig. S5B,C (original panels).**
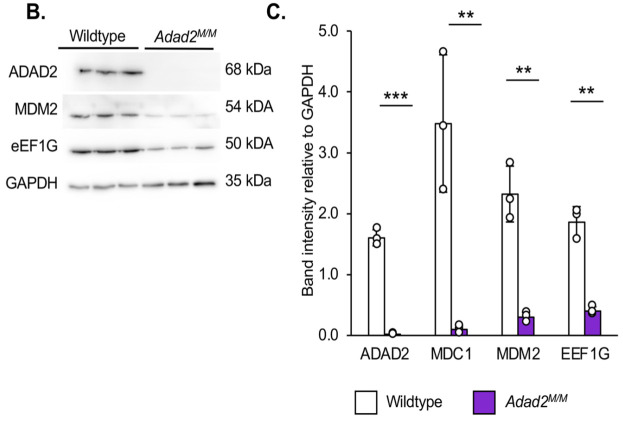
**Altered ribosome association similar to Mdc1 is observed in multiple transcripts but abundance and ribosome association is unaltered in heterochromatin remodeling genes in Adad2 mutants.** (B) Western blot against MDM2 and eEF1G in 21 dpp wildtype and mutant testes and (C) quantification of band intensity in B shows similar reduction of protein in spite of increased ribosome association. ADAD2 shown as genetic control (n=3/genotype). Plotted points represent individual samples.

In Fig. S5D, the GAPDH blot shown for 21 dpp samples was incorrect, and the eEF1G blot for 21 dpp samples was reused from Fig. S5B. The corrected and original panels are shown below. The figure legend has been updated to add details of data reuse.

**Fig. S5D (corrected panel).**
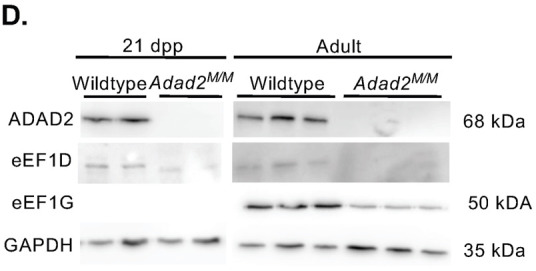
**Altered ribosome association similar to Mdc1 is observed in multiple transcripts but abundance and ribosome association is unaltered in heterochromatin remodeling genes in Adad2 mutants.** (D) Western blot against eEF1G and eEF1D in adult, and against eEF1D in 21 dpp wildtype and mutant testes (adult, n=3/genotype; 21 dpp, n=2/genotype). Abundance of eEF1G in 21 dpp wildtype and mutant testes can be seen in B. The ADAD2 genotype control blots and GAPDH control blots are also shown for the same protein sample panels in Fig. 1D, Fig. 3A, Fig. S5B and Fig. S6D.

**Fig. S5D (original panel).**
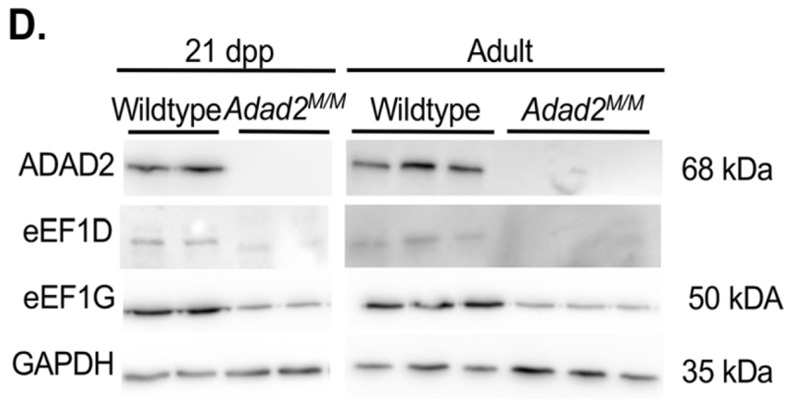
**Altered ribosome association similar to Mdc1 is observed in multiple transcripts but abundance and ribosome association is unaltered in heterochromatin remodeling genes in Adad2 mutants.** (D) Western blot against eEF1B complex components in adult (n=3/genotype) and 21 dpp (n=2/genotype) wildtype and mutant testes.

In Fig. S6A, reuse of the Total H3 loading control blot shown in Fig. 1E was not explained in the figure legend. The corrected legend reads:

(A) Western blot of 21 dpp whole testis histone lysate from wildtype and *Adad2^M/M^* samples (wildtype n=2; *Adad2^M/M^* n=3) showing increased γH2AX. Total H3 shown as loading control. The total H3 loading control blot is also shown for the same protein sample panel in Fig. 1E.

The original legend was:

(A) Western blot of 21 dpp whole testis histone lysate from wildtype and *Adad2^M/M^* samples (wildtype n=2; *Adad2^M/M^* n=3) showing increased γH2AX. Total H3 shown as loading control.

In Fig. S6D, the ADAD2, KU80 and GAPDH blots were vertically inverted. The corrected and original panels are shown below. The figure legend has been updated to add details of data reuse.

**Fig. S6D (corrected panel).**
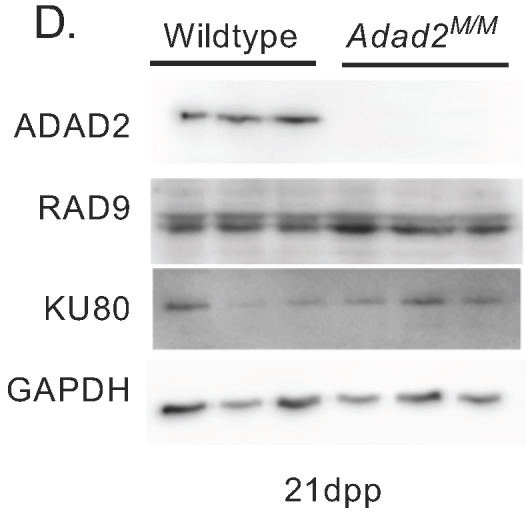
***Adad2^M/M^* spermatocytes show no evidence of aberrant DNA damage in spite of elevated γH2AX.** (D) Western blot against RAD9 and KU80 in 21 dpp wildtype and mutant testes showing lack of DNA damage repair pathway induction. ADAD2 and GAPDH shown as genotype and loading controls, respectively. The ADAD2 genotype control and GAPDH loading control blots are also shown for the same protein sample panel in Fig. 1D, Fig. 3A and Fig. S5B,D.

**Fig. S6D (original panel).**
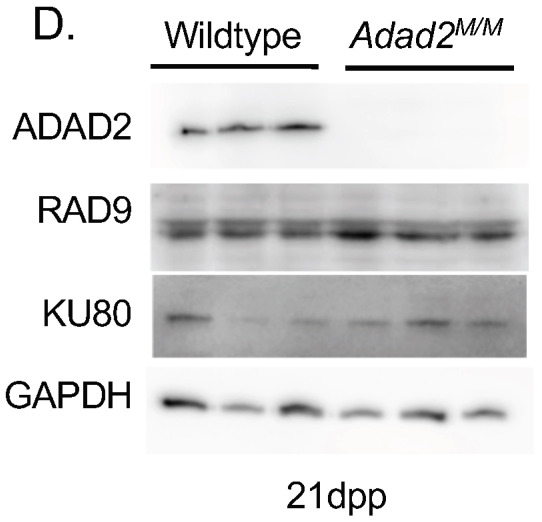
***Adad2^M/M^* spermatocytes show no evidence of aberrant DNA damage in spite of elevated γH2AX.** (D) Western blot against RAD9 and KU80 in 21 dpp wildtype and mutant testes showing lack of DNA damage repair pathway induction. ADAD2 and GAPDH shown as genotype and loading controls, respectively.

The authors wish to add further details to the Materials and Methods section to explain the experimental design of the western blot experiments. The following text has been added at the end of the first paragraph in the ‘Protein isolation and western blotting’ section of the Materials and Methods:

For each age, a single protein panel of biological replicates (*n*=3 per genotype for most analyses) was generated. Proteins were quantified, diluted to a concentration of 1 mg/ml and utilized for all western blot analyses. Sample order was held constant across all western blot analyses. For each protein panel, a single genotype control (ADAD2) and loading control (GAPDH) was generated. These controls are shown across multiple figures (Fig. 1D, Fig. 3A, Fig. S5B,D and Fig. S6D) to aid in reader analysis. In the case of eEF1D in 21 dpp testes (Fig. S5D), *n*=2 per genotype as the first wild-type sample and the last mutant sample were unavailable. The associated ADAD2 and GAPDH control blot images were cropped to remove these samples, and thus they align with the presented eEF1D samples.

The changes detailed above have been applied to the online full text and PDF versions of the article and the supplementary information. The authors apologise to readers for the many errors, which do not impact the conclusions of the article.

